# Treatment with LABA versus LAMA for stable COPD: a systematic review and meta-analysis

**DOI:** 10.1186/s12890-020-1152-8

**Published:** 2020-04-29

**Authors:** Akira Koarai, Hisatoshi Sugiura, Mitsuhiro Yamada, Tomohiro Ichikawa, Naoya Fujino, Tomotaka Kawayama, Masakazu Ichinose

**Affiliations:** 1 Department of Respiratory Medicine, Tohoku University Graduate School of Medicine, 1-1 Seiryo-machi, Aoba-ku, Sendai, 980-8574 Japan; 2 Division of Respirology, Neurology and Rheumatology, Department of Medicine, Kurume University School of Medicine, 67 Asahi-machi, Kurume, 830-0011 Japan

**Keywords:** Adverse events, Exacerbations, SGRQ, Trough FEV_1_, TDI

## Abstract

**Background:**

Inhaled bronchodilators including long-acting beta-agonist (LABA) and long-acting muscarinic antagonist (LAMA) play a central role in the treatment of stable chronic obstructive pulmonary disease (COPD). However, it is still unclear whether LABA or LAMA should be used for the initial treatment. Therefore, we conducted a systematic review and meta-analysis to evaluate the efficacy and safety of LABA versus LAMA in patients with stable COPD.

**Methods:**

We searched relevant randomized control trials (RCTs) with a period of treatment of at least 12 weeks and analyzed the exacerbations, quality of life, dyspnea score, lung function and adverse events as the outcomes of interest.

**Results:**

We carefully excluded unblinded data and identified a total of 19 RCTs (*N* = 28,211). LAMA significantly decreased the exacerbations compared to LABA (OR 0.85, 95% CI 0.74 to 0.98; *P* = 0.02). In St George’s Respiratory Questionnaire and transitional dyspnoea index score, there were no differences between LABA and LAMA treatment. Compared to LABA, there was a small but significant increase in the trough FEV_1_ after LAMA treatment (Mean difference 0.02, 95% CI 0.01 to 0.03, *P* = 0.0006). In the safety components, there was no difference in the serious adverse events between LABA and LAMA. However, LAMA showed a significantly lower incidence of total adverse events compared to LABA (OR 0.92, 95% CI 0.86 to 0.98; *P* = 0.02).

**Conclusion:**

Treatment with LAMA in stable COPD provided a significantly lower incidence of exacerbation and non-serious adverse events, and a higher trough FEV_1_ compared to LABA.

**Trial registration:**

(PROSPERO: CRD42019144764)

## Background

Chronic obstructive pulmonary disease (COPD) is currently the third leading cause of death in the world [1]. The most common symptoms include dyspnea, cough and sputum production, with the symptoms worsening during exacerbations. For the treatment of stable COPD patients, inhaled bronchodilators play a central role in reducing symptoms and exacerbations. Regular daily use of either a long-acting beta-agonist (LABA) or long-acting muscarinic antagonist (LAMA) has been shown to improve the lung function, dyspnea and health status and reduce exacerbations [2–4]. In addition, these bronchodilators improve exercise performance [5, 6]. Currently available LAMA comprises tiotropium, glycopyrronium, aclidinium and umeclidinium and LABA includes salmeterol, formoterol, indacaterol, vilanterol and olodaterol. Now, either LABA or LAMA is first used for the treatment of patients with stable COPD. According to the Global Initiative for Chronic Obstructive Lung Disease (GOLD) report 2019, there is no mention which bronchodilator, LABA or LAMA, is superior for the initial relief of the symptoms in the GOLD grade group A and B patients [7]. On the other hand, in group C and D patients that have experienced exacerbations of COPD, LAMA is more often recommended as a single initial therapy than LABA. However, this is only due to two head-to-head comparison studies which showed the superiority of tiotropium to salmeterol or indacaterol in preventing exacerbations [8, 9]. Therefore, it remains unclear which bronchodilator, LABA or LAMA, is more suitable for the initial treatment of stable COPD.

Until now, several systematic reviews have been reported for comparison between LABA and LAMA for the treatment of patients with stable COPD [10–13]. However, these reviews mainly compared tiotropium with salmeterol or indacaterol in up to six different studies. In the latest review, Chen, et al. evaluated 16 studies that included LABA (salmeterol, formoterol, indacaterol and olodaterol) and LAMA (tiotropium, glycopyrronium, aclidinium and umeclidinium), and showed the superiority of LAMA over LABA in the prevention of COPD exacerbation and fewer adverse events [13]. However, the analysis contained open labeled-tiotropium group or unblinded studies which could reduce the accuracy of the meta-analysis. Therefore, we conducted a systematic review and meta-analysis, not only excluding the unblinded data, but also adding several new trials.

To clarify whether LABA or LAMA is more beneficial for the initial treatment of patients with stable COPD, we searched relevant randomized control clinical trials and evaluated the efficacy and safety of LABA versus LAMA by measuring exacerbations, quality of life, dyspnea score, lung function and adverse events.

## Methods

### Search strategy and eligibility criteria

This systematic review and the meta-analysis were conducted according to the Preferred Reporting Items for Systematic Reviews and Meta-Analyses (PRISMA) guidance [14]. The study protocol was registered in the PROSPERO database (www.crd.york.ac.uk/prospero/; registration number: CRD42019144764). We firstly set outcomes from clinical importance and then performed a systematic research to acquire literature. We searched and identified randomized controlled trials in MEDLINE, Cochrane Central Register of Controlled Trials (CENTRAL), Pubmed and EMBASE databases on November 2018, using the search strategy provided in the on-line supplement. We also conducted a search of ClinicalTrials.gov (www.ClinicalTrials.gov). Only publications described in English were considered. As the inclusion criteria, participants had a diagnosis of stable COPD according to the GOLD report’s diagnostic criteria. Randomized controlled trials comparing LABA and LAMA were included if they evaluated any of our outcomes of interest with at least 12 weeks of treatment duration. In addition, studies with subgroup analysis of comparisons of LAMA/LABA combination therapy with the individual components were also included. Open labeled-tiotropium groups and unblinded studies were excluded from the analysis.

### Data collection and risk of bias assessment

At least two review authors (AK, MY, TI and NF) screened the titles and abstracts of all studies identified by the search strategy to check their eligibility. Next, full text assessments were performed to identify the studies for inclusion, and the data were retrieved from the eligible studies. At least two review authors (AK, MY, TI and NF) assessed the risk of bias in the eligible studies according to the recommendations in the Cochrane Handbook for Systematic Reviews of Interventions 5.1.0. If there were discrepancies in the data collection or assessment of the risk of bias, the review authors resolved the disagreements through discussion.

### Outcomes of interest

The included outcomes of interest in the current study were as follow: i) exacerbations (number of patients experiencing one or more exacerbations), ii) St George’s Respiratory Questionnaire (SGRQ) score change from the baseline, iii) transitional dysponea index (TDI) score change from the baseline, iv) trough forced expiratory volume in one second (FEV_1_) change from the baseline, and v) adverse events (total adverse events and serious adverse events).

### Statistical analysis

We analyzed dichotomous data as Mantel-Haenzsel odds ratios (OR) and continuous data as mean difference with 95% confidence intervals (CI) using Review Manager Software version 5.3 (Cochrane Library Software, Oxford, UK). We carefully checked whether the data were shown with standard deviation in each study and analyzed the data after the conversion from standard error to standard deviation if the data were shown as standard error. Inconsistency among studies was assessed by I_2_ statistic test. Publication bias was examined using funnel plots and assessed visually, and decided by Egger’s tests using R version 3.6.1 (The R Foundation for Statistical Computing Platform) if applicable. A subgroup analysis was performed in each drug included in the LABA and LAMA group and major adverse events. Quality of evidence was measured in accordance with the Grading of Recommendations Assessment, Development and Evaluation (GRADE), and absolute estimates of effect for the outcomes were also evaluated [15].

## Results

### Characteristics of selected studies

**Fig. 1:**
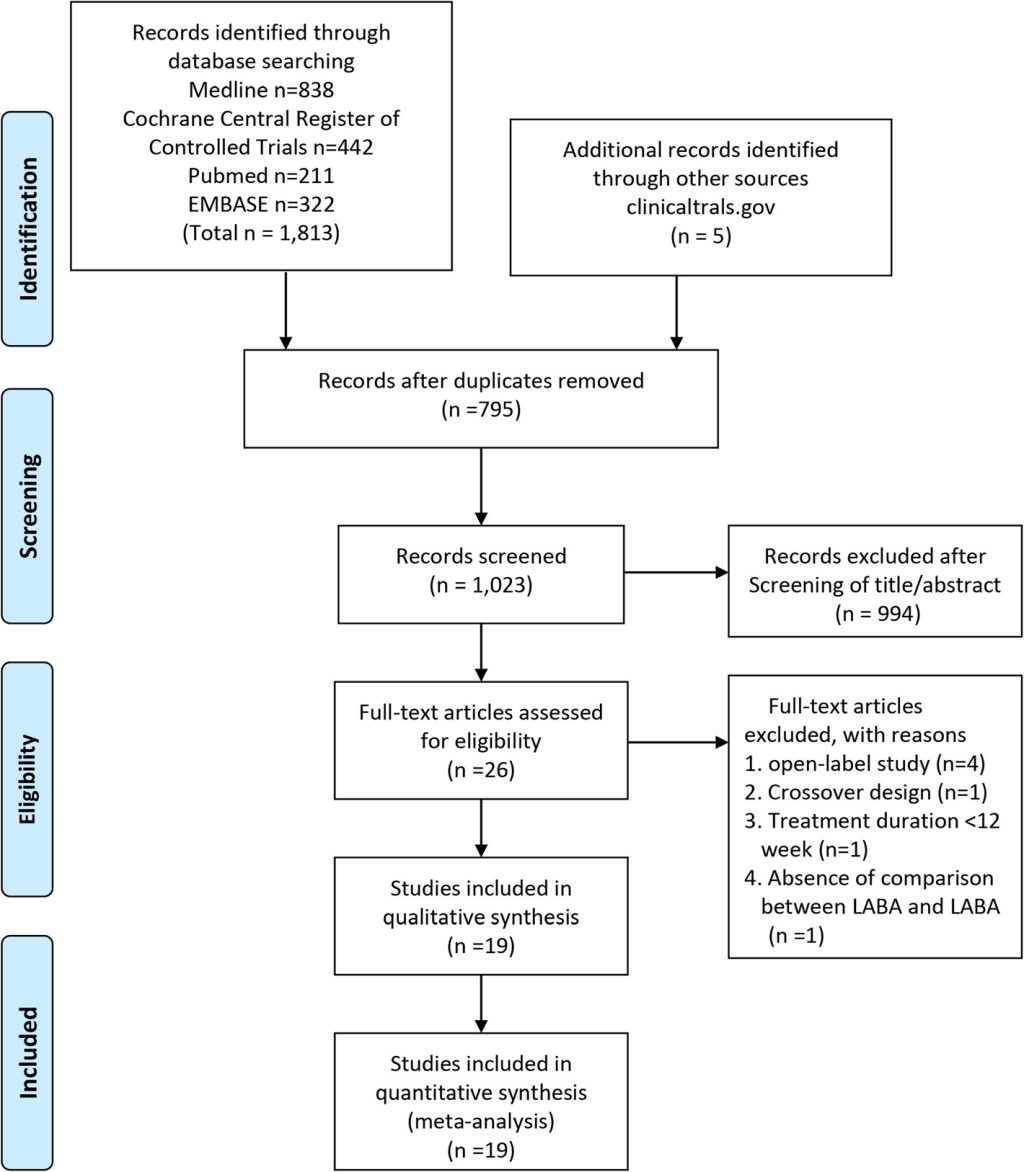
Flow diagram of study selection

Search strategies yielded 1023 candidate studies, excluding duplicates. After full-text assessment, we excluded 7 trials [16–22] and finally identified a total of 19 RCTs eligible for the meta-analysis (Fig. 1 and additional file: Table S1). These studies were published from 2002 to 2018 and their characteristics are summarized in Table 1 and additional file: Table S2.

**Table 1:** Characteristics of included studies

study	Treatment (μg)	Number of subjects	Duration (weeks)	Key inclusion criteria	Male (%)	Mean age (years)	Baseline FEV_1_ (%predicted)
Donohue 2002	Tiotoropium 18 Salmeterol 100	623	24	%FEV_1_ < 60% > 40 yrs., > 10PY	74.6	64.0	40.2
Brusasco 2003	Tiotoropium 18 Salmeterol 100	807	24	%FEV_1_ ≤ 65%, > 40 yrs., > 10PY	76.2	64.0	42.9
Briggs 2005	Tiotoropium 18 Salmeterol 100	653	12	%FEV_1_ < 60%, > 40 yrs., > 10PY	66.4	64.4	37.7
Buhl 2011 INTENSITY study	Tiotoropium 18 Indacaterol 150	1598	12	%FEV_1_ 30–80%, > 40 yrs., > 10PY	69.0	63.5	54.4
Vogelmeier 2011 POET study	Tiotoropium 18 Salmeterol 100	7376	52	%FEV_1_ ≤ 70%, ≥ 40 yrs., ≥ 10PY, Ex (+)	74.6	62.9	49.3
Bateman 2013 SHINE study	Glycopyrronium 50^a^ Indacaterol 150^a^	1435	26	%FEV_1_ ≥ 30%, < 80% ≥ 40 yrs., ≥ 10PY	75.4	63.9	53.7
Decramer 2013 INVIGORATE	Tiotoropium 18 Indacaterol 150	3439	52	%FEV_1_ > 30%, ≤ 50% ≥ 40 yrs., ≥ 10PY, Ex (+)	77.0	64.0	40.5
Donohue 2013	Umeclidinium 62.5^a^ Vilanterol 25^a^	839	24	%FEV_1_ ≤ 70% mMRC ≥2, ≥ 40 yrs., ≥ 10PY	69.0	63.3	47.5
Celli 2014	Umeclidinium 125^a^ Vilanterol 25^a^	811	24	%FEV_1_ ≤ 70% mMRC ≥2, ≥ 40 yrs., ≥ 10PY	66.0	63.0	48.7
Decramer 2014	Tiotoropium 18^a^ Vilanterol 25^a^	417	24	%FEV_1_ ≤ 70% mMRC ≥2, ≥ 40 yrs., ≥ 10PY	68.0	62.9	47.7
D’Urzo 2014 AUGMENT study	Aclidinium 800^a^ Formoterol 24^a^	669	24	%FEV_1_ ≥ 30%, < 80% mMRC ≥2, ≥ 40 yrs., > 10PY	56.0	64.2	53.3
Singh 2014 ACLIFORM-COPD	Aclidinium 800^a^ Formoterol 24^a^	769	24	%FEV_1_ < 70% mMRC ≥2, ≥ 40 yrs., > 10PY	66.5	63.2	54.0
Buhl 2015 TOnado 1 and 2	Tiotoropium 2.5/5^a^ Olodaterol 5^a^	2071	52	%FEV_1_ > 30%, < 80% mMRC ≥2, ≥ 40 yrs., > 10PY	73.4	64.1	50.0
Mahler 2015 FLIGHT1 and 2	Glycopyrrolate 31.2^a^ Indacaterol 55^a^	1022	12	%FEV_1_ > 30%, < 80% mMRC ≥2, ≥ 40 yrs., > 10PY	64.8	63.6	54.5
Mahler 2016 GEM3 study	glycopyrrolate 31.2 Indacaterol 75	511	52	%FEV_1_ ≥ 30%, < 80% mMRC> 2, ≥ 40 yrs., ≥ 10PY	57.2	63.2	53.1
D’Urzo 2017 AUGMENT study	Aclidinium 800^a^ Formoterol 24^a^	669	52	%FEV_1_ ≥ 30%, < 80% ≥ 40 yrs., ≥ 10PY	56.0	64.2	53.3
Hanania 2017 PINNACLE-3	Glycopyrronium 36^a^ Formoterol 19.2^a^	1772	52	%FEV_1_ ≥ 30%, < 80% or %FEV_1_ < 30% & FEV_1_ ≥ 750 ml, 40–80 yrs	55.8	62.8	51.3
Martinez 2017 PINNACLE-1, −2	Glycopyrronium 36^a^ Formoterol 19.2^a^	1776	24	%FEV_1_ ≥ 30%, < 80% or %FEV_1_ < 30% & FEV_1_ ≥ 750 ml, 40–80 yrs	55.8	62.8	51.3
Lipworth 2018 PINNACLE-4	Glycopyrronium 36^a^ Formoterol 19.2^a^	954	24	%FEV_1_ < 80% 40–80 yrs., ≥ 10PY	74.1	64.1	54.4

Definition of abbreviations: *FEV*_*1*_ forced expiratory volume in 1 s, *yrs* years, *PY* pack-years

Ex(+): at least one exacerbation in the previous year; mMRC: modified Medical Research Council dyspnoea scale

^a^individual components of subgroup analysis in comparison of LAMA/LABA combination therapy

Eight studies evaluating the effects of tiotropium compared with salmeterol (four studies) [9, 23–25], indacaterol (two studies) [8, 26], vilanterol (one study) [27] and olodaterol (one study) [28], six studies evaluating the effects of glycopyrronium or glycopyrrolate compared with indacaterol (three studies) [29–31] and formoterol (three studies) [32–34], two studies evaluating the effects of umeclidinium compared with vilanterol [35, 36], and three studies evaluating the effects of aclidinium compared with formoterol were included [37–39]. Two extension trials (D’Urzo 2017 and Hanania 2017) [32, 39] from other studies and one trial (Mahler 2016) [31] for safety assessment were included. Therefore, data from these trials were carefully selected not to count the same patients twice (Additional file: Table S2). All the studies were randomized double to quadra blinded controlled trials. Twelve studies’ data were derived from the individual components of subgroup analysis in the comparison of the LAMA/LABA combination therapy. Participants were at least 40 years of age, current or ex-smokers with a smoking history of 10 pack-years or more, and the severity of the disease was moderate to severe. Treatment period was from 12 to 52 weeks.

### Risk of bias

Six studies had unclear risk in the random sequence generation and allocation concealment (selection bias). The risks of blinding of participants and personnel (performance bias) were all low and unclear risk in the blinding of outcome assessment (detection bias) was included in seven studies. Two trials had high risk in the incomplete outcome data (attrition bias). In other biases, all studies were determined to contain unclear risk because the sponsors were all pharmaceutical companies (additional file: Table S3 and Table S4). Possible publication bias assessed by funnel plots was seen in serious adverse events (additional file: Fig. S1), but there was no significant difference in the Egger’s tests (data not shown).

### Outcome assessments

#### Exacerbations

**Fig. 2:**
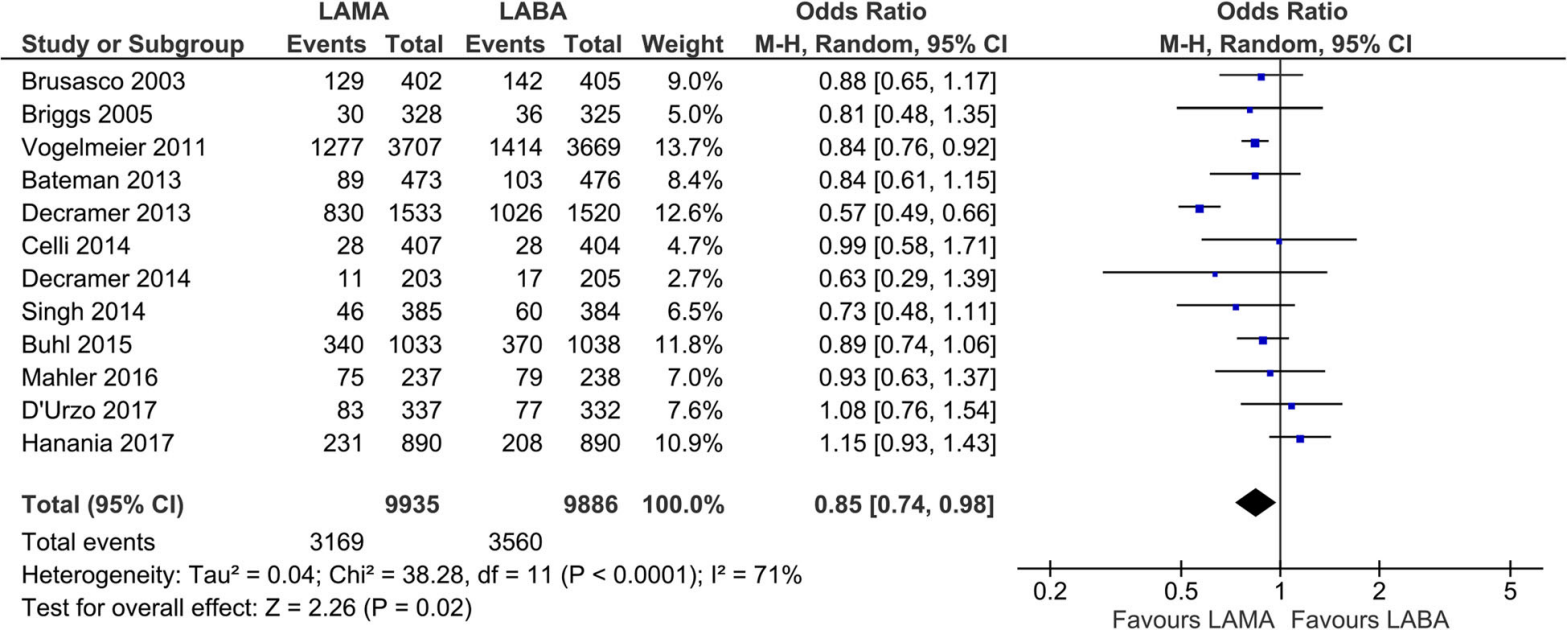
Efficacy of long-acting bronchodilators on exacerbations

Twelve studies with 19,821 participants were included for the evaluation of exacerbations. Based on these studies with 12 to 52 weeks of observation, 31.9% (3169/9935) of the patients treated with LAMA experienced one or more exacerbations compared with 36.0% (3560/9886) of those with LABA (Fig. 2). In the patients treated with LAMA, there was a significant decrease in the number of exacerbations compared to those treated with LABA (OR 0.85, 95% CI 0.74 to 0.98; *P* = 0.02; I_2_ = 71%; Fig. 2). In the subgroup analysis, there were significant reductions in the number of exacerbations when comparing tiotropium to salmeterol in three studies (OR 0.84, 95% CI 0.77 to 0.92; *P* = 0.0001; I_2_ = 0%; additional file: Fig. S2) or to indacaterol in one study (OR 0.57, 95% CI 0.49 to 0.66; *P* < 0.00001; I_2_: not applicable; additional file: Fig. S2). With any other drugs, there were no significant differences between LABA and LAMA.

#### SGRQ and TDI score

**Fig. 3:**
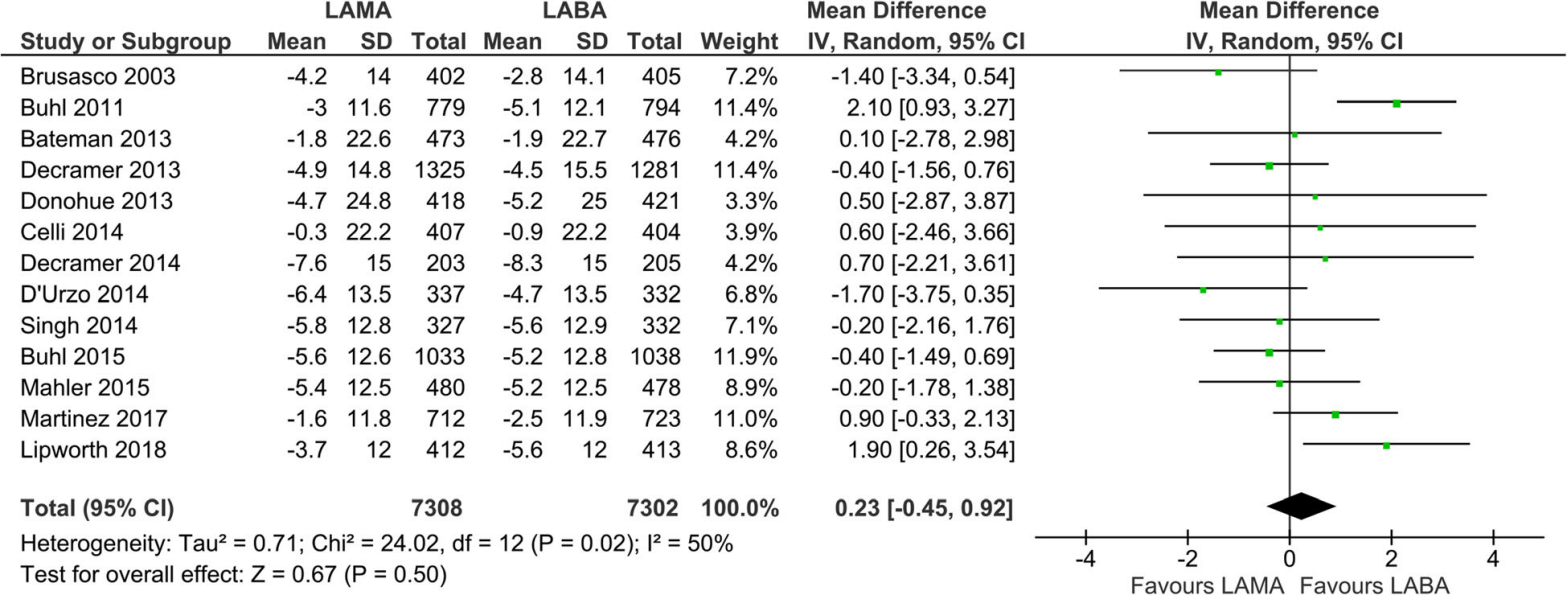
Efficacy of long-acting bronchodilators on quality of life: change from baseline in SGRQ score

**Fig. 4:**
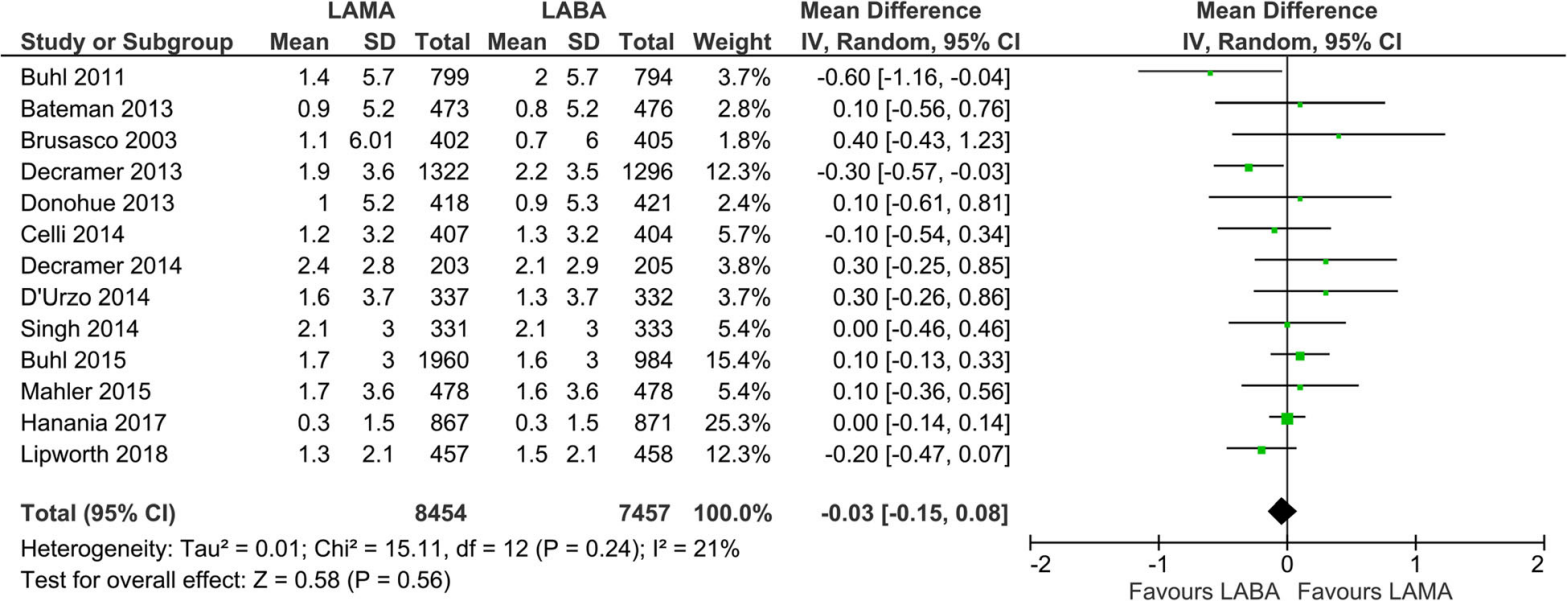
Efficacy of long-acting bronchodilators on symptoms: change from baseline in TDI score

Thirteen studies with 14,610 participants were included for evaluation of the SGRQ score. The mean SGRQ score change from the baseline was − 4.2 ± 15.5 (SD) with LAMA (*N* = 7308) and − 4.3 ± 15.7 (SD) with LABA treatment (*N* = 7302) (Fig. 3). The mean change in the SGRQ score was not statistically different between LABA and LAMA (mean difference 0.23, 95% CI: − 0.45 to 0.92, *P* = 0.50, I_2_ = 50%; Fig. 3). In the subgroup analysis, only one drug of LABA, formoterol, was significantly superior to glycopyrronium in the SGRQ score change from the baseline evaluated by two studies (mean difference 1.26, 95% CI 0.28 to 2.24; *P* = 0.01; I_2_ = 0%; Additional file: Fig. S3). Concerning the TDI score, thirteen studies with 15,911 participants were evaluated, and the mean TDI score change from the baseline was 1.4 ± 3.8 (SD) with LAMA (*N* = 8454) and 1.5 ± 3.9 (SD) with LABA treatment (*N* = 7457) (Fig. 4). There was no statistical difference in the TDI score change from the baseline between the LAMA and LABA treatment (mean difference − 0.03, 95% CI − 0.15 to 0.08, *P* = 0.56, I_2_ = 21%) (Fig. 4). In the subgroup analysis, only one LABA, indacaterol, was significantly superior to tiotropium in the TDI score change from the baseline in two studies (mean difference − 0.36, 95% CI − 0.60 to − 0.11; *P* = 0.004; I_2_ = 0%; additional file: Fig. S4).

#### Trough FEV_1_.

**Fig. 5:**
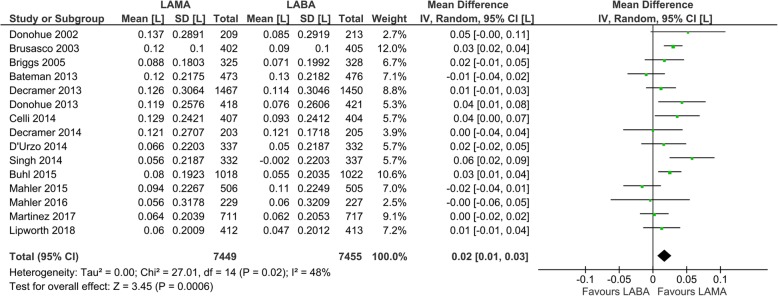
Efficacy of long-acting bronchodilators on trough FEV_1_: change from baseline

Fifteen studies with 14,904 participants were included for evaluation of the trough FEV_1_. The mean trough FEV_1_ change from the baseline was 0.098 ± 0.240 (SD) with LAMA (*N* = 7449) and 0.081 ± 0.240 (SD) with LABA treatment (*N* = 7455) (Fig. 5). Compared to LABA, there was a small but significant increase in the trough FEV_1_ with LAMA treatment (mean difference 0.02, 95% CI 0.01 to 0.03, *P* = 0.0006, I_2_ = 48%: Fig. 5). However, this difference was less than the minimal clinically important difference of 0.05 to 0.10 L [40]. In the subgroup analysis, there were significant increases in the trough FEV_1_ with LAMA compared with LABA treatment when comparing tiotropium to salmeterol in three studies (mean difference 0.03, 95% CI 0.02 to 0.04; *P* < 0.00001; I_2_ = 0%; additional file: Fig. S5) or olodaterol in one study (mean difference 0.03, 95% CI 0.01 to 0.04; P = 0.004; I_2_; not applicable; additional file: Fig. S5), and also when comparing umeclidinium to vilanterol in two studies (mean difference 0.04, 95% CI 0.02 to 0.06; *P* = 0.001; I_2_ = 0%; additional file: Fig. S5).

#### Adverse events

**Fig. 6:**
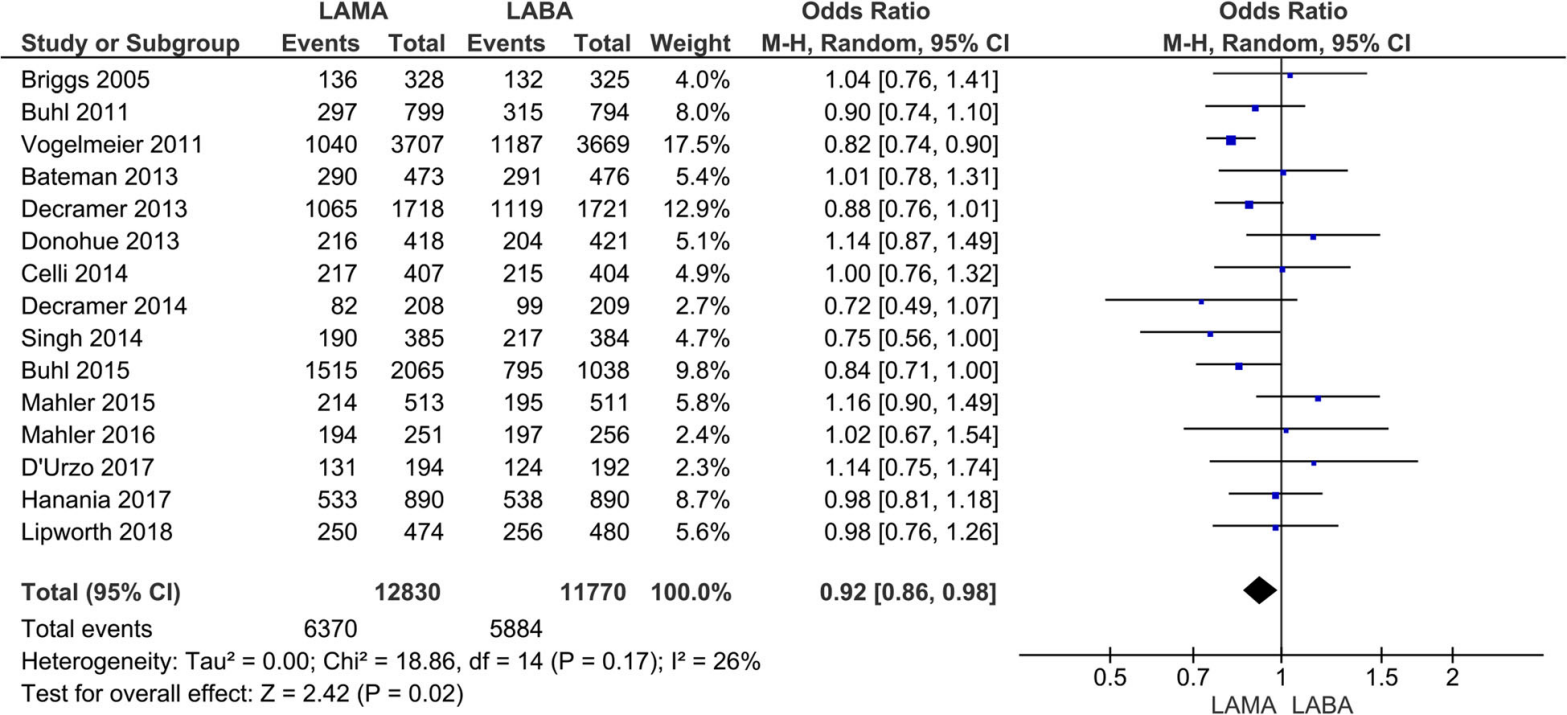
Efficacy of long-acting bronchodilators on total adverse events

**Fig. 7:**
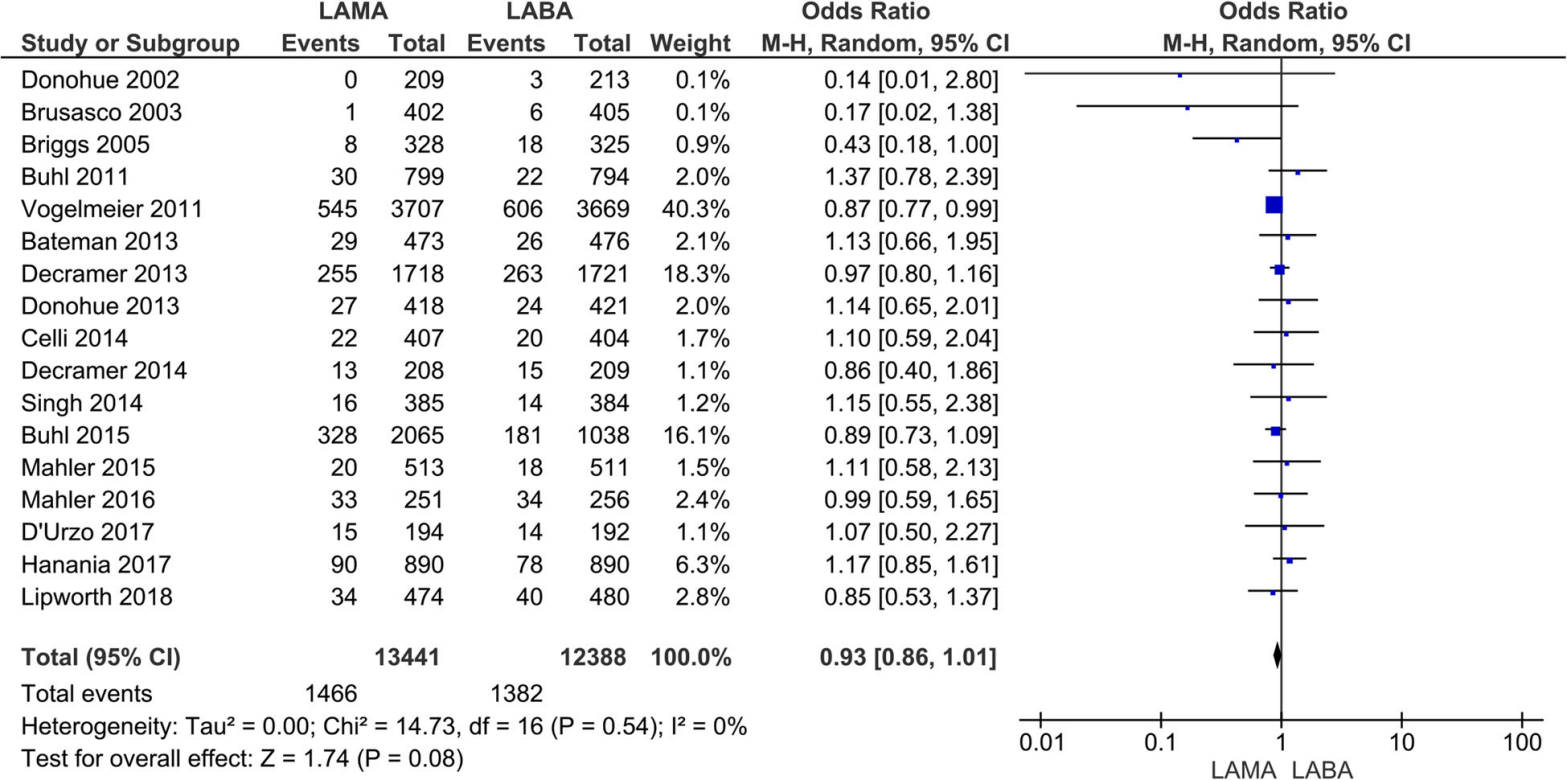
Efficacy of long-acting bronchodilators on serious adverse events

Fifteen studies with 24,600 participants were included for the evaluation of total adverse events. The total adverse events were 6370 (*N* = 12,830) with LAMA and 5884 (*N* = 11,770) with LABA treatment (Fig. 6). In the patients treated with LAMA, there was a significant decrease in the number of total adverse events compared to those treated with LABA (OR 0.92, 95% CI 0.86 to 0.98; *P* = 0.02; I_2_ = 26%; Fig. 6). In the subgroup analysis, there was a significant decrease in the number of total adverse events with LAMA compared with LABA treatment when comparing tiotropium to indacaterol in two studies (OR 0.88, 95% CI 0.79 to 0.99; *P* = 0.04; I_2_ = 0%; additional file: Fig. S6). Concerning serious adverse events, the events were 1466 (*N* = 13,441) with LAMA and 1382 (N = 12,388) with LABA treatment in seventeen studies (Fig. 7). There was no significant difference between the LAMA and LABA treatment (OR 0.93, 95% CI 0.86 to 1.01; *P* = 0.08; I_2_ = 0%; Fig. 7) and the sub-analysis also did not detect significant differences between each drug (additional file: Fig. S7).

The details of adverse events were evaluated in 13 studies with frequency threshold for reporting at from 1.5 to 5%. Dry mouth, one of the major adverse events with LAMA, was reported until 2005 in three studies [23–25] and the incidence was from 5 to 10% (data not shown). Thereafter, dry mouth was not included as an adverse event. In the sub-analysis of total adverse events, main adverse events were COPD-related events, such as symptom worsening and COPD exacerbations, nasopharingitis and upper respiratory tract infections. For the most part, there was no significant difference between the LAMA and LABA treatment. Only in lower respiratory tract infection and hypertension with an incidence of less than 4% was the frequency significantly lower with LAMA than that with LABA treatment (lower respiratory tract infection: OR 0.62, 95% CI 0.39 to 0.98; *P* = 0.04; I_2_ = 29%; hypertension: OR 0.67, 95% CI 0.52 to 0.86; *P* = 0.002; I_2_ = 2%, additional file: Table S5 and Fig.S8A, S8B). In major adverse cardiovascular event (MACE), non-MACE and mortality, there was no significant difference between the LAMA and LABA treatments (additional file: Table S5 and Fig.S8C).

In addition, we evaluated the details of adverse events in indacaterol, especially, COPD-related adverse events and cough because indacaterol showed a significantly higher incidence than tiotropium in the current meta-analysis. Compared to non-Indacaterol LABA treatment, indacaterol showed a higher incidence of cough, but not of COPD-related adverse events, than LAMA (OR 0.59, 95% CI 0.46 to 0.75; *P* < 0.0001; I_2_ = 7%, additional file: Fig.S9A, S9B).

## Discussion

In the current systematic review and meta-analysis, we demonstrated that mono-treatment with LAMA in stable COPD provided a significantly lower incidence of exacerbations and total adverse events, and higher trough FEV_1_ compared to LABA. In the SGRQ and TDI score and serious adverse events, there were no significant differences between LABA and LAMA treatment. The overall quality of evidence was moderate for all outcomes (additional file: Table S6). These results confirm that LAMA is a more suitable treatment than LABA for patients of COPD with previous experience of exacerbations. In addition, LAMA might be also a better treatment than LABA for stable COPD patients due to its higher trough FEV_1_ and lower risk of non-serious adverse events.

Until now, four systematic reviews have been reported for comparison between LABA and LAMA for the treatment of patients with stable COPD [10–13]. In 2012, Chong*,* et al. evaluated six studies to compare tiotropium with LABA including salmeterol, formoterol and indacaterol, and demonstrated the superiority of tiotropium over LABA in the prevention of COPD exacerbation and a lower rate of non-fatal serious adverse events [10]. On the other hand, Rodrigo*,* et al. evaluated five studies comparing indacaterol with tiotropium or other LABA and demonstrated that indacaterol significantly achieved a minimal clinically important difference in the TDI and SGRQ score compared to tiotropium [11]. In 2015, Kim*,* et al. re-evaluated four studies of comparison between indacaterol and tiotropium [12]. They demonstrated that there were no differences in trough FEV_1_ and SGRQ scores between LABA and LAMA treatment, but indacaterol showed higher incidences of cough and worsening of COPD in the adverse events than tiotropium [12]. In 2017, Chen, et al. re-evaluated 16 studies including LABA (salmeterol, formoterol, indacaterol and olodaterol) and LAMA (tiotropium, glycopyrronium, aclidinium and umeclidinium), and demonstrated the superiority of LAMA to LABA in reducing exacerbations with fewer adverse events [13]. These reviews have shown that LAMA is superior to LABA in reducing the risk of exacerbations with fewer adverse events, but not in the SGRQ and TDI score and trough FEV_1_. In the current study, we showed that LAMA decreased the incidence of exacerbation and total adverse events in COPD patients compared to LABA, which confirms the results from previous reviews. Concerning the trough FEV_1_, we firstly demonstrated the superiority of LAMA treatment to LABA. This difference might be due to the selection of studies in which our current meta-analysis excluded unblinded data and newly included several studies.

Concerning exacerbations, we confirmed the superiority of LAMA treatment to LABA in reducing the rate of exacerbation of COPD in patients with stable COPD. In the evaluation of twelve trials, there was a high grade of inconsistency (I_2_ = 71%).This might be due to differences in the inclusion criteria for participants who previously experienced exacerbation. In the current analysis, only two trials (Vogelmeier, et al. 2011 and Decramer, et al. 2013) included a history of exacerbations for the inclusion criteria [8, 9]. These two trials both demonstrated the significant superiority of LAMA (tiotropium) treatment to LABA (salmeterol or indacaterol) in reducing the risk of exacerbations. In addition, the inconsistency might be also due to differences in the drugs. In the subgroup analysis, the superiority of LAMA to LABA was only detected in tiotropium compared to salmeterol or indacaterol. However, there were no differences in the incidence of exacerbations between any other drugs, which were not administered to patients with a history of exacerbations. Further studies in patients with a history of exacerbations are needed to clarify this point. Overall, in the meta-analysis of COPD exacerbation, most of the studies showed no difference between LAMA and LABA treatment, but the total results were reversed when two large studies that targeted the exacerbation were included. Therefore, there remains a limitation to assuming the superiority of LAMA to LABA in the exacerbation to whole drugs.

In trough FEV_1_, we firstly demonstrated the superiority of LAMA treatment to LABA. The superiority was also shown in the subgroup analysis when comparing not only tiotropium to salmeterol or olodaterol, but also umeclidinium to vilanterol. The actual difference of 10 to 30 mL is very small and below the 50 to 100 mL level of minimal clinically important difference for which the index is usually used for a comparison with placebo [40–42]. In fact, in our present analysis, the difference in the trough FEV_1_ did not cause a significant change in the patient’s QOL and symptoms evaluated by the SGRQ and TDI score. However, the small difference is nearly similar to that of the trough FEV_1_ between ICS/LABA/LAMA and LABA/LAMA therapy [mean difference 38.05 ml 95%CI 22.04–54.06 ml] [43], and this difference may affect some clinical courses in patients with a more severe grade of COPD, because a higher level of trough FEV_1_ is related to a lower risk of exacerbations and reduced annual decline of FEV_1_ [44, 45]. At present, there are no clinical data to support this possibility, but the superiority of LAMA to LABA in the trough FEV_1_ might affect the selection of a bronchodilator when patients are first treated with LABA or LAMA.

In our current results of the SGRQ and TDI score, there were no significant differences between LABA and LAMA treatment. However, in the subgroup analysis, indacaterol was significantly superior to tiotropium in the TDI score, which is consistent with a previous result [11]. In the SGRQ score, we firstly demonstrated that formoterol was more effective than glycopyrronium. These results are inconsistent with the inferiority of LABA to LAMA in the trough FEV_1_ in the current results [44, 45], but these discrepancies might be explained by the earlier or higher peak bronchodilatory effect of LABA compared to LAMA in the comparison between these drugs [16, 26, 33, 34]. In the current systematic review, the early or peak bronchodilatory effect was out of scope because of the small amount of uniform data. Further trials and analysis are needed.

Concerning the safety components, our current analysis showed a small but significantly lower incidence of total adverse events in LAMA compared to LABA. In the subgroup analysis, indacaterol showed a significantly higher incidence than tiotropium, which is consistent with a previous result [12]. In a previous review, Kim, et al showed that indacaterol caused a higher incidence of cough and worsening of COPD than tiotropium [12]. In the current analysis, we confirmed that indacaterol showed a higher incidence of cough than LAMA treatment. Cough is a common complaint associated with indacaterol at the concentration of 150 μg [8, 18]. Cough-associated indacaterol is known to occur just after inhalation and lasts for only six seconds without any negative effects on the safety or efficacy [18]. A lower concentration of indacaterol has been reported to reduce this complaint [46]. In fact, in our current analysis, at a lower concentration (55 or 75 μg) of indacaterol, there was no difference in the total adverse events between indacaterol and glycopyrrolate [30, 31]. Also, in the subgroup analysis, LABA treatment showed a small but significantly higher incidence of lower respiratory tract infection and hypertension than LAMA. Lower respiratory tract infection is one of the causes of COPD exacerbation, therefore the higher incidence with LABA is consistent with the higher frequency of COPD exacerbation with LABA than with LAMA. Concerning the higher incidence of hypertension with LABA, this could be explained as a beta-agonist related adverse effect [47]. However, this result should be cautiously considered because it is mainly mentioned in only one study by Buhl 2015 [28] and several studies suggested that LABA treatment reduces the incidence of hypertension in COPD [48, 49]. In the serious adverse events including MACE, non-MACE and mortality, there was no difference between LABA and LAMA treatment, which is consistent with previous reviews [10–13].

There are several limitations in our current systematic review and meta-analysis. First, in the current analysis, the evaluated patients were all less than 80% of %FEV_1_; therefore, the results are not supported for mild COPD patients. Secondly, there were still not enough trials with some drugs to perform subgroup analysis. Third, we did not evaluate the physical activity including exercise performance, which is one of the important therapeutic targets in COPD patients, due to the lack of a trial that met our selection criteria.

## Conclusions

In the current systematic review and meta-analysis, we demonstrated that treatment with LAMA in stable COPD provided a significantly lower incidence of exacerbations and non-serious adverse events, and a higher trough FEV_1_ compared to LABA. The overall quality of evidence was moderate for all outcomes. These results suggest that LAMA might be a more preferable treatment than LABA, not only for patients with previous experience of exacerbations, but also for patients with any grade of COPD due to the bronchodilatory effect on trough FEV_1_ and lower risk of non-serious adverse events.

## Supplementary information

**Additional file 1: Table S1.** List of and reason why studies have been excluded from the analysis. **Table S2.** Characteristics of included studies for the analysis of each outcome. **Table S3.** Assessment of risk of bias. **Table S4.** Details for the risk bias assessment. **Table S5.** Details of adverse events (%). **Table S6.** Summary of findings for the main comparison. **Figure S1** Funnel plots for exacerbations, SGRQ score, TDI score, Trough FEV1, total adverse events and severe adverse events (all studies). **Figire S2.** Subanalysis for exacerbations by each drug. **Figu S3.** Subanalysis for SGRQ score by each drug. **Figure S4** Subanalysis for TDI sore by each drug. **Figire S5** Subanalysis for trough FEV1 by each drug. **Figire S6.** Subanalysis for total adverse events by each drug. **Figu S7.** Subanalysis for serious adverse events by each drug. **Figure S8** Subanalysis for adverse events. **Figure S9.** Subanalysis for adverse events (Indacaterol vs non-Indacaterol).

## Data Availability

Source data and material will be made available from the corresponding author upon reasonable request.

## Abbreviations

CENTRAL: Cochrane Central Register of Controlled Trials
CI: Confidence intervals
COPD: Chronic obstructive pulmonary disease
FEV_1_: Forced expiratory volume in one second
GOLD: Global Initiative for Chronic Obstructive Lung Disease
LABAs: Long-acting beta-agonists
LAMAs: Long-acting muscarinic antagonist
OR: Odds ratios
PRISMA: Preferred Reporting Items for Systematic Reviews and Meta-Analyses
SD: Standard deviation
SGRQ: St George’s Respiratory Questionnaire
TDI: Transitional dyspnea index
