# Supplementary material for: Treatment with LABA versus LAMA for stable COPD: a systematic review and meta-analysis
Source: BMC Pulm Med. 2020 Apr 29;20:111. doi: 10.1186/s12890-020-1152-8 (PMC7191827; doi:10.1186/s12890-020-1152-8)
Supplement: Supplementary file 1 — Additional file 1: Table S1. List of and reason why studies have been excluded from the analysis. Table S2. Characteristics of included studies for the analysis of each outcome. Table S3. Assessment of risk of bias. Table S4. Details for the risk bias assessment. Table S5. Details of adverse events (%). Table S6. Summary of findings for the main comparison. Figure S1 Funnel plots for exacerbations, SGRQ score, TDI score, Trough FEV1, total adverse events and severe adverse events (all studies). Figire S2. Subanalysis for exacerbations by each drug. Figu S3. Subanalysis for SGRQ score by each drug. Figure S4 Subanalysis for TDI sore by each drug. Figire S5 Subanalysis for trough FEV1 by each drug. Figire S6. Subanalysis for total adverse events by each drug. Figu S7. Subanalysis for serious adverse events by each drug. Figure S8 Subanalysis for adverse events. Figure S9. Subanalysis for adverse events (Indacaterol vs non-Indacaterol). [file 12890_2020_1152_MOESM1_ESM.docx]

**Treatment with LABA versus LAMA for stable COPD: a systematic review and meta-analysis**

*Akira Koarai, M.D., Ph.D.^1^, Hisatoshi Sugiura, M.D., Ph.D.^1^, Mitsuhiro Yamada, M.D., Ph.D.^1^, Tomohiro Ichikawa M.D., Ph.D.^1^, Naoya Fujino M.D., Ph.D.^1^, Tomotaka Kawayama, M.D., Ph.D. ^2^ and Masakazu Ichinose, M.D., Ph.D. ^1^

^1^ Department of Respiratory Medicine, Tohoku University Graduate School of Medicine, 1-1 Seiryo-machi, Aoba-ku, Sendai 980-8574, Japan.

^2^ Division of Respirology, Neurology and Rheumatology, Department of Medicine, Kurume University School of Medicine, 67 Asahi-machi, Kurume 830-0011, Japan.

**Additional file**

**Search strategy for MEDLINE**

| # | Searches |
| --- | --- |
| 1 | Lung Diseases, Obstructive/ |
| 2 | exp Pulmonary Disease, Chronic Obstructive/ |
| 3 | emphysema*.mp. |
| 4 | (chronic* adj3 bronchiti*).mp. |
| 5 | (chronic* adj3 (pulmonary or lung* or airway* or airfow* or bronch* or respirat*)).mp. |
| 6 | COPD.mp. |
| 7 | COAD.mp. |
| 8 | COBD.mp. |
| 9 | AECB.mp. |
| 10 | or/1-9 |
| 11 | clinical trial.pt. |
| 12 | (randomized or randomised).ab,ti. |
| 13 | placebo.ab,ti. |
| 14 | dt.fs. |
| 15 | randomly.ab,ti. |
| 16 | trial.ab,ti. |
| 17 | groups.ab,ti. |
| 18 | or/11-17 |
| 19 | Animals/ |
| 20 | Humans/ |
| 21 | 19 not (19 and 20) |
| 22 | 18 not 21 |
| 23 | Adrenergic beta-2 Receptor Agonists.sh. |
| 24 | (long* adj beta* adj agonist*).mp. |
| 25 | salmeterol*.mp. |
| 26 | formoterol*.mp. |
| 27 | indacaterol*.mp. |
| 28 | QAB-149.mp. |
| 29 | vilanterol*.mp. |
| 30 | GW642444.mp. |
| 31 | olodaterol*.mp. |
| 32 | BI 1744 CL.mp. |
| 33 | tulobuterol*.mp. |
| 34 | bambuterol*.mp. |
| 35 | clenbuterol*.mp. |
| 36 | or/23-35 |
| 37 | Muscarinic Antagonists.sh. |
| 38 | (muscarinic* adj antagonist*).mp. |
| 39 | LAMA.ab,ti. |
| 40 | tiotropium*.mp. |
| 41 | Spiriva.mp. |
| 42 | glycopyrronium*.mp. |
| 43 | NVA237.mp. |
| 44 | Seebri.mp. |
| 45 | umeclidinium*.mp. |
| 46 | GSK573719.mp. |
| 47 | Incruse.mp. |
| 48 | aclidinium*.mp. |
| 49 | LAS34273.mp. |
| 50 | Turdorza.mp. |
| 51 | Eklira.mp. |
| 52 | or/37-51 |
| 53 | 10 and 22 and 36 and 52 |

**Search strategy for CENTRAL, Pubmed, EMBASE and ClinicalTrials.gov**

#1 COPD OR chronic obstructive lung disease

#2 (tiotropium OR glycopyrronium OR umeclidinium OR aclidinium) AND (salmeterol OR formoterol OR indacaterol OR vilanterol OR olodaterol OR bambuterol OR clenbuterol)

#3 randomized OR randomised

#1 AND #2 AND #3

**Table S1 List of and reason why studies have been excluded from the analysis.**

| **Study** | **Reason for exclusion** |
| --- | --- |
| Vogelmeier C, et al: Formoterol mono- and combination therapy with tiotropium in patients with COPD: a 6-month study. Respiratory Medicine 2008; 102: 1511-20 | Open-label study |
| Barnes PJ, et al: Integrating indacaterol dose selection in a clinical study in COPD using an adaptive seamless design. Pulmonary Pharmacology & Therapeutics 2010; 23: 165-71 | Treatment duration <12 week |
| Donohue JF, et al: Once-daily bronchodilators for chronic obstructive pulmonary disease: indacaterol versus tiotropium. American Journal of Respiratory & Critical Care Medicine 2010; 182: 155-62 | Open-label study |
| Wedzicha JA, et al: Analysis of chronic obstructive pulmonary disease exacerbations with the dual bronchodilator QVA149 compared with glycopyrronium and tiotropium (SPARK): a randomised, double-blind, parallel-group study. The Lancet Respiratory Medicine 2013; 1: 199-209 | Absence of comparison between LABA and LABA |
| Maltais F, Singh S, Donald AC, et al: Effects of a combination of umeclidinium/vilanterol on exercise endurance in patients with chronic obstructive pulmonary disease: two randomized, double-blind clinical trials.　Therapeutic Advances in Respiratory Disease 2014; 8: 169-81 | Crossover study |
| Watz H, et al: Indacaterol improves lung hyperinflation and physical activity in patients with moderate chronic obstructive pulmonary disease--a randomized, multicenter, double-blind, placebo-controlled study. BMC Pulmonary Medicine 2014; 14: 158 | Open-label study |
| Hoshino M, Ohtawa J: Computed tomography assessment of airway dimensions with combined tiotropium and indacaterol therapy in COPD patients. Respirology 2014; 19: 403-10 | Open-label study |

| **Table S2** Characteristics of included studies for the analysis of each outcome | | | | | | | | | | | |
| --- | --- | --- | --- | --- | --- | --- | --- | --- | --- | --- | --- |
| study | Treatment　(µg) | Duration  (weeks) | Country (N) | Primary  outcome | Secondary  outcome | Ex | SG  RQ | TDI | FEV_1_ | AE | SAE |
|  |  |  |  |  | |  |  |  |  |  |  |
| Donohue　2002 | Tiotoropium 18  Salmeterol 100 | 24 | 12 | Lung function, Symptoms  Adverse events | |  |  |  | ✔ |  | ✔ |
|  |  |  |  |  | |  |  |  |  |  |  |
| Brusasco 2003 | Tiotoropium 18  Salmeterol 100 | 24 | 18 | Exacerbations, Lung function Symptoms, Adverse events | | ✔ | ✔ | ✔ | ✔ |  | ✔ |
|  |  |  |  |  |  |  |  |  |  |  |  |
| Briggs 2005 | Tiotoropium 18  Salmeterol 100 | 12 | 8 | Lung function | Exacerbations,  Adverse events | ✔ |  |  | ✔ | ✔ | ✔ |
|  |  |  |  |  |  |  |  |  |  |  |  |
| Buhl 2011  INTENSITY study | Tiotoropium 18  Indacaterol 150 | 12 | 41 | Lung function | Symptoms, Adverse events |  | ✔ | ✔ |  | ✔ | ✔ |
|  |  |  |  |  |  |  |  |  |  |  |  |
| Vogelmeier 2011  POET study | Tiotoropium 18  Salmeterol 100 | 52 | 25 | Exacerbations | Adverse events | ✔ |  |  |  | ✔ | ✔ |
|  |  |  |  |  |  |  |  |  |  |  |  |
| Bateman　2013  SHINE study | Glycopyrronium 50*  Indacaterol 150* | 26 | 19 | Lung function | Symptoms Exacerbations | ✔ | ✔ | ✔ | ✔ | ✔ | ✔ |
|  |  |  |  |  |  |  |  |  |  |  |  |
| Decramer 2013  INVIGORATE | Tiotoropium 18  Indacaterol 150 | 52 | 41 | Lung function | Exacerbations | ✔ | ✔ | ✔ | ✔ | ✔ | ✔ |
|  |  |  |  |  |  |  |  |  |  |  |  |
| Donohue 2013 | Umeclidinium　62.5*  Vilanterol　25* | 24 | 13 | Lung function | Symptoms |  | ✔ | ✔ | ✔ | ✔ | ✔ |
|  |  |  |  |  |  |  |  |  |  |  |  |
| Celli 2014 | Umeclidinium　125*  Vilanterol　25* | 24 | 14 | Lung function | Symptoms | ✔ | ✔ | ✔ | ✔ | ✔ | ✔ |
|  |  |  |  |  |  |  |  |  |  |  |  |
| Decramer 2014 | Tiotoropium 18*  Vilanterol　25* | 24 | 9 | Lung function | Lung function | ✔ | ✔ | ✔ | ✔ | ✔ | ✔ |
|  |  |  |  |  |  |  |  |  |  |  |  |
| D'Urzo 2014  AUGMENT study | Aclidinium 800*  Formoterol 24* | 24 | 4 | Lung function | Symptoms |  | ✔ | ✔ | ✔ |  |  |
|  |  |  |  |  |  |  |  |  |  |  |  |
| Singh 2014  ACLIFORM-COPD | Aclidinium 800*  Formoterol 24* | 24 | 22 | Lung function | Symptoms | ✔ | ✔ | ✔ | ✔ | ✔ | ✔ |
|  |  |  |  |  |  |  |  |  |  |  |  |
| Buhl 2015  TOnado 1 and 2 | Tiotoropium 2.5/5*  Olodaterol 5* | 52 | 26/24 | Lung function  Symptoms | Lung function  Symptoms | ✔ | ✔ | ✔ | ✔ | ✔ | ✔ |
|  |  |  |  |  |  |  |  |  |  |  |  |
| Mahler 2015  FLIGHT1 and 2 | Glycopyrrolate 31.2* Indacaterol　 55* | 12 | 8/9 | Lung function  Symptoms | Lung function  Symptoms |  | ✔ | ✔ | ✔ | ✔ | ✔ |
|  |  |  |  |  |  |  |  |  |  |  |  |
| Mahler 2016  GEM3 study | glycopyrrolate 31.2  Indacaterol 75 | 52 | 1 | Adverse events | Lung function  Symptoms Exacerbations | ✔ |  |  | ✔ | ✔ | ✔ |
|  |  |  |  |  |  |  |  |  |  |  |  |
| D'Urzo 2017  AUGMENT study | Aclidinium 800*  Formoterol 24* | 52 | 2 | Adverse events | Adverse event | ✔ |  |  |  | ✔ | ✔ |
|  |  |  |  |  |  |  |  |  |  |  |  |
| Hanania 2017  PINNACLE-3 | Glycopyrronium 36*  Formoterol 19.2* | 52 | 3 | Lung function | Symptoms Lung function | ✔ |  | ✔ |  | ✔ | ✔ |
|  |  |  |  |  |  |  |  |  |  |  |  |
| Martinez 2017  PINNACLE-1, -2 | Glycopyrronium 36*  Formoterol 19.2* | 24 | 3/1 | Lung function | Lung function  Symptoms |  | ✔ |  | ✔ |  |  |
|  |  |  |  |  |  |  |  |  |  |  |  |
| Lipworth 2018  PINNACLE-4 | Glycopyrronium 36*  Formoterol 19.2* | 24 | 11 | Lung function | Symptoms Lung function |  | ✔ | ✔ | ✔ | ✔ | ✔ |
|  |  |  |  |  |  |  |  |  |  |  |  |
| Definition of abbreviations: Ex = exacerbations; FEV_1_ = forced expiratory volume in 1 second; AE = adverse events;  SAE = serious adverse events; ✔ = included studies  * individual components of subgroup analysis in comparison of LAMA/LABA combination therapy | | | | | | | | | | | |


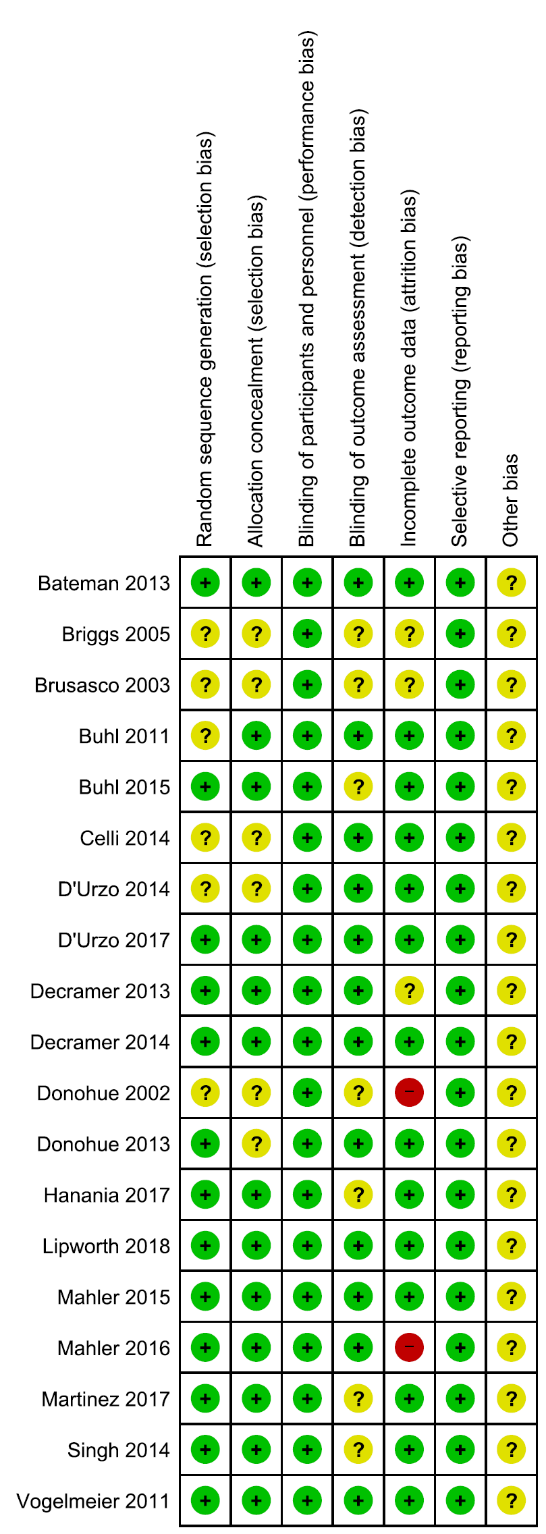
**Table S3 Assessment of risk of bias.**


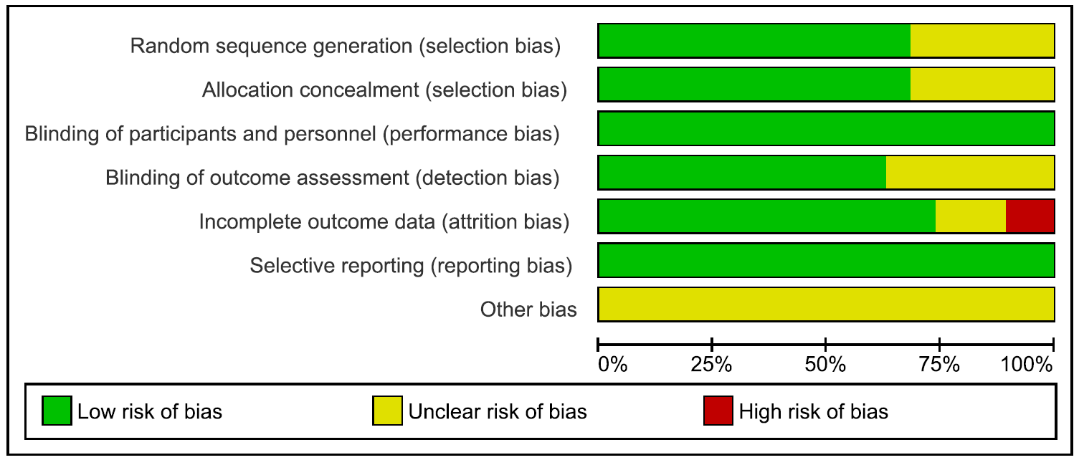

**Table S6 Summary of findings for the main comparison.**

**Fig. S1 Funnel plots for exacerbations, SGRQ score, TDI score, Trough FEV_1_, total adverse events and severe adverse events (all studies).**

**Exacerbations**

**SGRQ score**

**TDI score**

**Trough FEV_1_**

**Total adverse events**

**Serious adverse events**


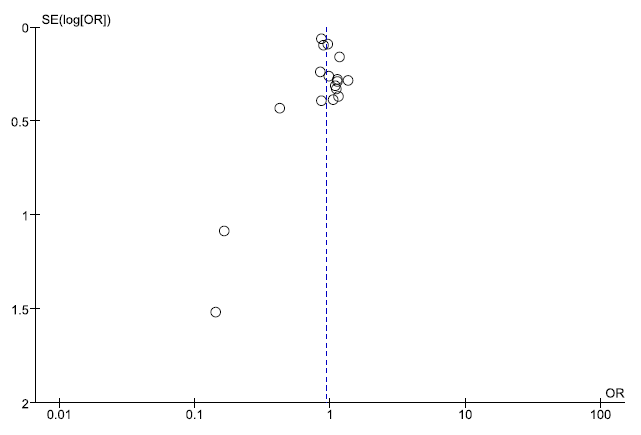


**Fig. S2 Subanalysis for exacerbations by each drug**

**Fig. S3 Subanalysis for SGRQ score by each drug**

**Fig. S4 Subanalysis for TDI sore by each drug**


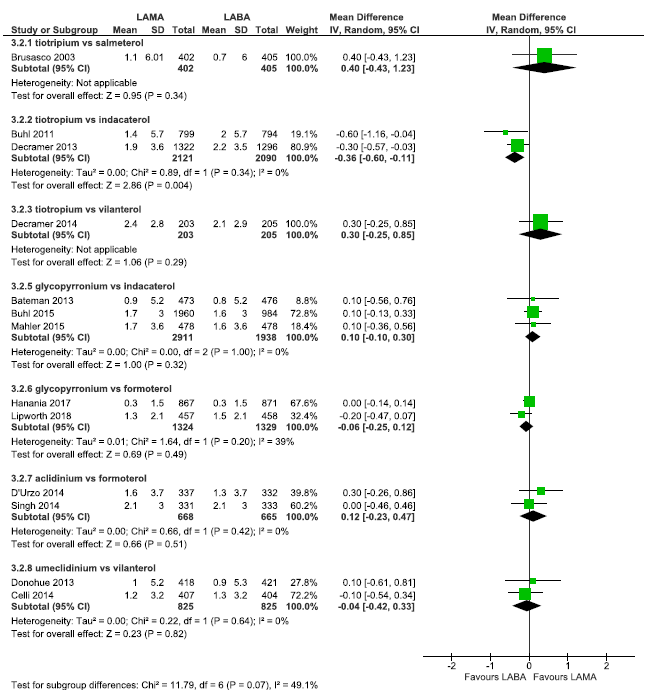


**Fig. S5 Subanalysis for trough FEV_1_ by each drug**

**Fig. S6 Subanalysis for total adverse events by each drug**


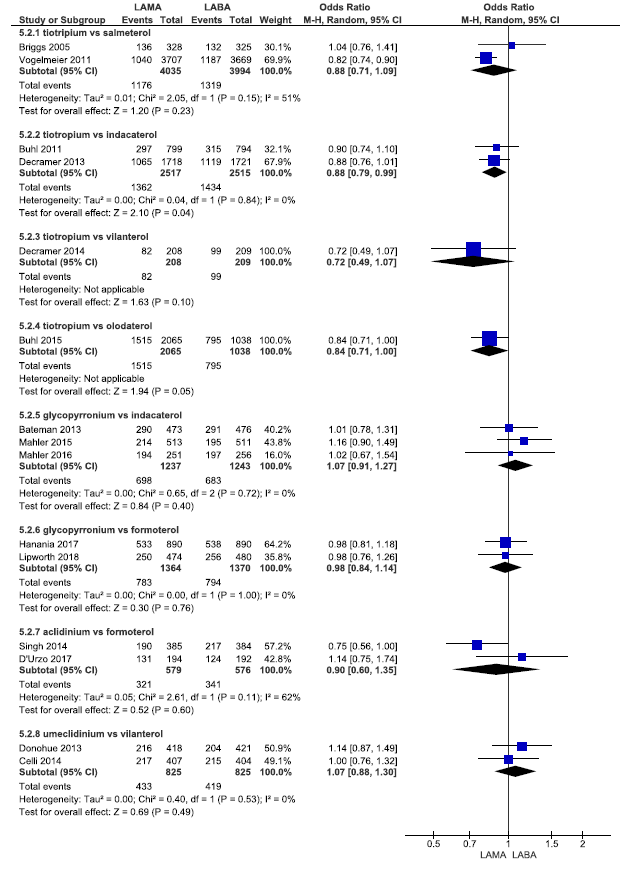


**Fig. S7 Subanalysis for serious adverse events by each drug**


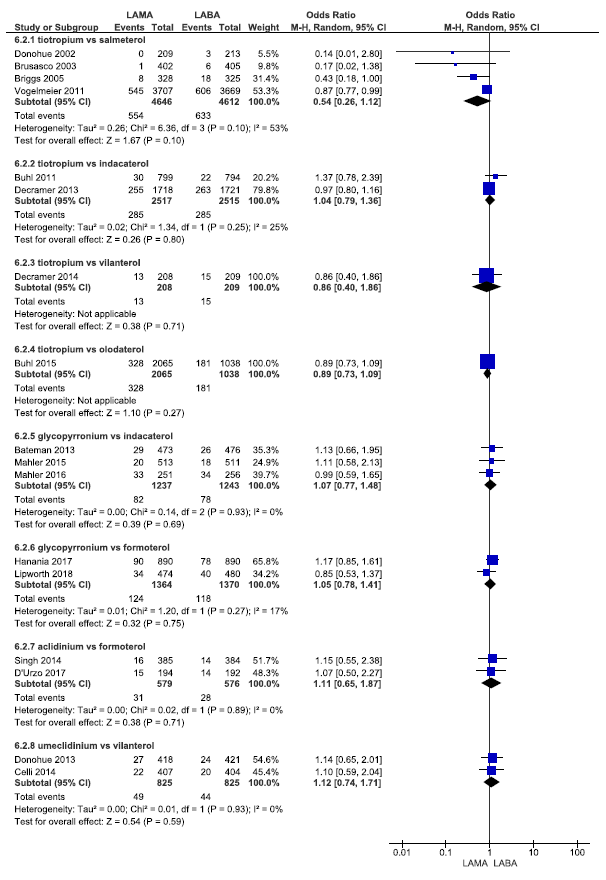


**Fig. S8 Subanalysis for adverse events**

**A) Lower respiratory tract infection**

**B) Hypertention**

**C) Mortality**

**Fig. S9 Subanalysis for adverse events (Indacaterol vs non-Indacaterol)**

**A) COPD**

**B) Cough**
